# Study of the Carrier-Aided Thin Film Electrode Array Design for Cochlear Insertion

**DOI:** 10.3390/mi9050206

**Published:** 2018-04-27

**Authors:** Yuchen Xu, Chuan Luo, Fan-Gang Zeng, John C. Middlebrooks, Harrison W. Lin, Zheng You

**Affiliations:** 1 State Key Laboratory of Precision Measurement Technology and Instrument, Tsinghua University, Beijing 100083, China; xyc13@mails.tsinghua.edu.cn; 2 Department of Precision Instrument, Tsinghua University, Beijing 100083, China; 3 Beijing Laboratory for Biomedical Detection Technology and Instrument, Tsinghua University, Beijing 100083, China; 4 Departments of Biomedical Engineering, Otolaryngology—Head and Neck Surgery and Center for Hearing Research, University of California Irvine, Irvine, CA 92697, USA; fzeng@uci.edu (F.-G.Z.); middlebj@uci.edu (J.C.M.); harrison.lin@uci.edu (H.W.L.)

**Keywords:** cochlear implant, thin film electrode array, Parylene, carrier-aided insertion

## Abstract

The micro-fabricated thin film electrode array (TFEA) has been a promising design for cochlear implants (CIs) because of its cost-effectiveness and fabrication precision. The latest polymer-based cochlear TFEAs have faced difficulties for cochlear insertion due to the lack of structural stiffness. To stiffen the TFEA, dissolvable stiffening materials, TFEAs with different structures, and TFEAs with commercial CIs as carriers have been invested. In this work, the concept of enhancing a Parylene TFEA with Kapton tape as a simpler carrier for cochlear insertion has been proved to be feasible. The bending stiffness of the Kapton-aided TFEA was characterized with an analytical model, a finite element model, and a cantilever bending experiment, respectively. While the Kapton tape increased the bending stiffness of the Parylene TFEA by 10^3^ times, the 6-μm-thick TFEA with a similar Young’s modulus, as a polyimide, in turn significantly increased the bending stiffness of the 170-μm-thick Kapton carrier by 60%. This result indicated that even the TFEA is ultra-flexible and that its bending stiffness should not be neglected in the design or selection of its carrier.

## 1. Introduction

The cochlear implant (CI) has been one of the most successful neural prostheses to date. As a neural interface, contemporary CI electrode arrays consist of 12–26 electrodes spanning 18–31 mm in length and adopt a manual assembly method that leads to a wire-bundle array structure [1]. Such design features can be further improved since the performance and accessibility of CIs have been limited by a relatively small number of electrodes and the labor-intensive assembly method of arrays [2]. Micro-fabrication method has been a potential solution to the present dilemma of CI electrode arrays with both a higher fabrication precision and a lower cost brought by its scalability, promoting the development of micro-electromechanical (MEMS) CI thin film electrode arrays (TFEAs) [3,4]. Earlier versions of CI TFEAs adopted a silicon-based design [5,6], the rigid structure of which has been proven unfit for spiral-shaped cochlear insertion. To adjust to the anatomical features of the cochlea and to reduce insertion trauma, polymer-based flexible TFEAs were then developed [7]. Limitation for these flexible TFEAs was the ultra-low stiffness, which constrained the TFEA to be advanced into the perilymph-filled cochlea.

To mechanically enhance a TFEA, one approach is to combine coating or filling materials that dissolve after implantation. Such attempts include the application of polyethylene glycol, used by Takeuchi et al. to fill the Parylene micro-channel on an array for structural enhancement [8], carboxy-methylcellulose, used by Takeuchi et al. to encapsulate a Parylene-platinum neural probe during insertion [9], or maltose, used by Zhuolin et al. to coat a polyimide neural probe for brain penetration [10]. The use of dissolvable materials is ideal when the TFEA only needs to be stiffened temporarily and does not require a fixed shape after insertion. Another approach is to include designed structures to stiffen the TFEA or to allow for the use of an insertion tool. Yu et al. developed a nickel-based TFEA with enhanced mechanical robustness to penetrate a rat’s hippocampus for neural recording [11]. Jonathan et al. designed a thermal-formed 3D-sheath neural probe, enabling the assistance of a stainless steel or tungsten microwire during insertion [12,13]. Johnson and Wise designed a Parylene CI TFEA with backing rings to increase its bending stiffness from 0.2 kN·μm^2^ to 1.4 kN·μm^2^ (a 600% increase), which also enabled the use of an insertion stylet [7,14]. These designed stiffening structures, however, require extra fabrication steps such as lithography, dry film resist bonding, and post-thermal molding. Apart from the above-mentioned approaches, cochlear TFEAs specifically need to sustain a curved shape to “hug” the cochlear modiolus and deliver a stimulating current after insertion. In this case, another widely adopted approach is to adhere the TFEA onto a pre-shaped carrier, which involves little fabrication efforts for both the carrier and the TFEA-carrier assembly. Arcand et al. adhered a silicon-based TFEA onto a fluidic PET chamber [15]. Iverson et al. adhered a flexible polyimide TFEA onto an insertion test device (ITD, MED-EL Corp., Innsbruck, Austria) [16]. With computed tomography (CT) scans, such carrier-aided designs were found to significantly improve the insertion of TFEA. However, contemporary TFEA carriers have not yet developed a design methodology. The most successful TFEA carrier reported was a duplication of the silicone-filled CI array with no electrode or electric wire [16]. Such design was based on the assumption that an ultra-flexible TFEA has negligible effects on the mechanical properties of the carrier. In this case, the design of TFEA carrier was separated from the TFEA.

In this work, a proof-of-concept study focusing on the mechanical properties of carrier-aided TFEA was presented with a Parylene TFEA adhered onto a laminate Kapton tape (Dupont Corp., Wilmington, DE, USA). Bending stiffness of the Kapton-aided TFEA (TFEA-Kapton) was studied for its close relation to the bending and buckling force during cochlear insertion, which leads to insertion trauma. Section 2 demonstrates the components and preparations procedures of the TFEA-Kapton. Section 3 characterizes the bending stiffness before and after adhering the TFEA onto the Kapton tape with an analytical model, a finite element model (FEM), and a cantilever beam bending experiment, severally. Limitations of the present work and major findings useful for future TFEA carrier design are discussed in Section 4.

## 2. Preparations of TFEA-Kapton

**Figure 1:**
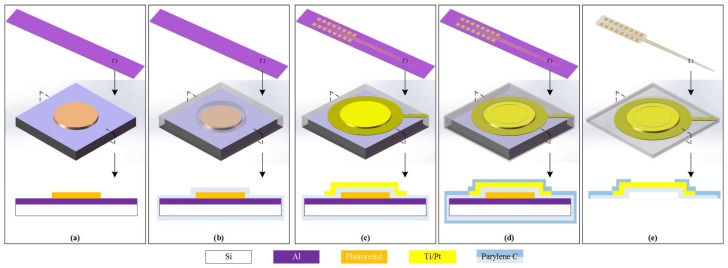
Schematics of the fabrication process, the three rows demonstrates the whole thin film electrode array (TFEA) structure, one electrode, and its cross-section view, respectively: (**a**) deposition of 500-nm aluminum and lithography of 2.2 μm AZ GXR-601 (AZ Electronic Materials Co., Wiesbaden, Germany); (**b**) deposition of 5 μm Parylene C; (**c**) Lift-off of 20-nm titanium and 200-nm platinum, respectively; (**d**) deposition of 1 μm Parylene C, Parylene patterning with AZ 4620, and O_2_ plasma etching; (**e**) TFEA release with fuming nitric acid.

A Parylene thin film electrode array was custom-designed to meet the geometrical constraints of the human cochlea, with a length of 21 mm and a substrate–metal–insulation structure. Biocompatible materials were chosen for the TFEA, with Parylene C being the 5-μm-thick substrate and 1-μm-thick insulation layer, titanium (Ti) being the 20-nm-thick adhesion layer, and platinum (Pt) being the 200-nm-thick connecting wires and electrodes. To deliver a stimulating current, the TFEA had 15 electrodes with a surface area of 1.7 × 10^4^ μm^2^ and an electrode interval of 1 mm, evenly spanned on the array. The TFEA was made by a micro-fabrication method, consisting of a sequence of photoresist transferring, lift-off, oxygen plasma etching, and fuming nitric acid release, as shown in Figure 1a–e.

**Figure 2:**
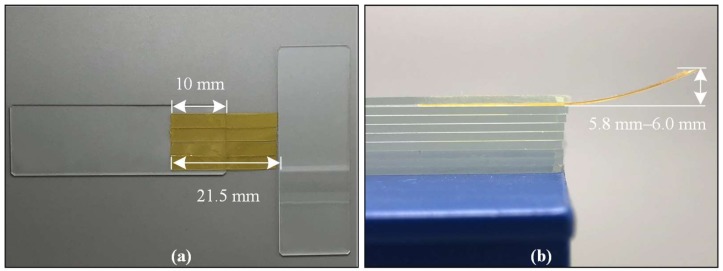
(**a**) The four Kapton tapes peeled off from the roll with the same 21.5 mm length (carved by a fine scalpel) and the clamped edge being aligned; (**b**) The upward vertical bending distance measured by a ruler.

One-mil (25.4-μm) double-sided Kapton tape with a single liner (sold by Xinshi corp., Guangdong, China), a commercially available tape consisting of a 25-μm-thick polyimide film as the backing layer, two 35-μm-thick silicone adhesive layers at each side, and a 75-μm-thick polyethylene terephthalate (PET) as the release liner [17] were pre-shaped for cochlear insertion. The Kapton tape was chosen as the TFEA carrier in this work for the following reasons: (1) It provided the electrical insulation (3.9 F/m at 1 kHz) needed for TFEA stimulation; (2) It was cost-effective and widely available; (3) It had a planar shape similar to the TFEA and can be easily pre-shaped to stiffen the TFEA. Additionally, it had an upward pre-bending shape after the tape roll was peeled off, adjusting to the spiral cochlear structure. The variability of different Kapton tapes’ curvature was characterized as shown in Figure 2. Given the same length (21.5 mm) of Kapton tape peeled off from the roll, and the same 10-mm-long basal edge clamped and aligned by glass slides, the 4 tested tapes had various vertical bending distances with a difference of ~3.3% (minimum distance: 5.8 mm, maximum distance: 6.0 mm).

**Figure 3:**
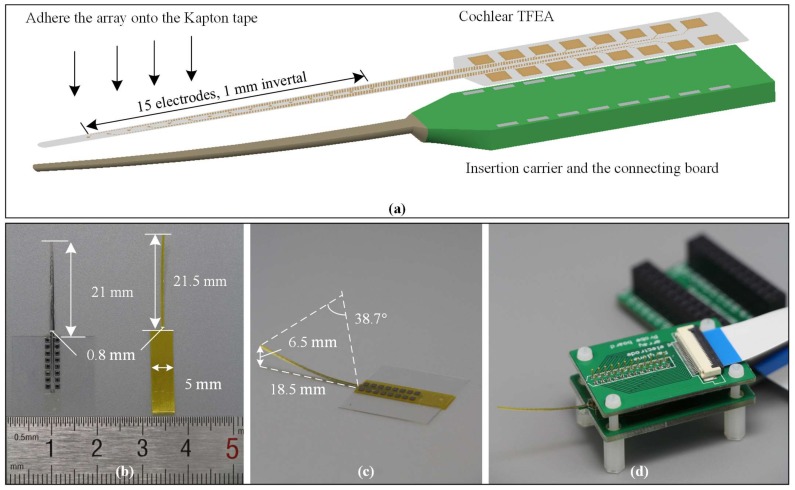
(**a**) Schematic of the TFEA; (**b**–**d**) Preparation of the TFEA and TFEA-Kapton: (**b**) TFEA sample and pre-shaped Kapton tape carrier; (**c**) adhering the TFEA onto the Kapton tape carrier; (**d**) electrical connection of the TFEA-Kapton onto a print circuit board.

The TFEA-Kapton was made with the following procedures to achieve the carrier-aided design in Figure 3a: (1) The basal part of a TFEA was affixed onto a piece of cover glass (18 mm × 18 mm × 0.15 mm) with a drop of deionized water, which vacated the air beneath the TFEA and adhered the TFEA firmly onto the glass with atmospheric pressure; (2) Kapton tape was adhered onto a flat surface and manually carved with a fine scalpel following the dimensions of the TFEA as shown in Figure 3b; (3) The apical part of the Kapton tape (0.8 mm in width) was covered by the matte surface of a glass slide; (4) The cover glass carrying the TFEA was pressed onto the basal part of the Kapton tape (5 mm in width), with the apical part of the TFEA placed onto the polished surface of the glass slide. By gently pushing the glass slide away from the Kapton tape along the lengthwise direction of the TFEA, the apical part of the TFEA fell onto the apical part of the Kapton tape and adhered to it, as shown in Figure 3c. The curved TFEA-Kapton had a central angle of 38.7°, which was less than the insertion depth angle of cochlea but adequate for a lateral-wall inserted TFEA [18]. The TFEA-Kapton was installed onto a print circuit board with spring-loaded pins for electrical connections, as shown in Figure 3d.

The TFEA-Kapton’s functionality for mechanical enhancement and cochlear stimulation was further characterized with an in vivo test in a cat model, showing an insertion depth of ~7 mm, a threshold of ~126 μA, and a dynamic range of ~8 dB (stimulus: 123-μs/phase biphasic single pulse). The electrical integrity of TFEA electrodes was verified before and after the in vivo test via an impedance test in the 0.01 mol/L phosphate buffer saline (1 × PBS). The electrical characters of the TFEA-Kapton will be available in an upcoming article focusing on the design of the CI Parylene TFEA.

## 3. Characterizations of Bending Stiffness

### 3.1. Theoretical Analyses of the TFEA-Kapton’s Bending Stiffness

To investigate the mechanical properties of the TFEA-Kapton, its bending stiffness was analyzed in an analytical model and an FEM, respectively.

**Figure 4:**
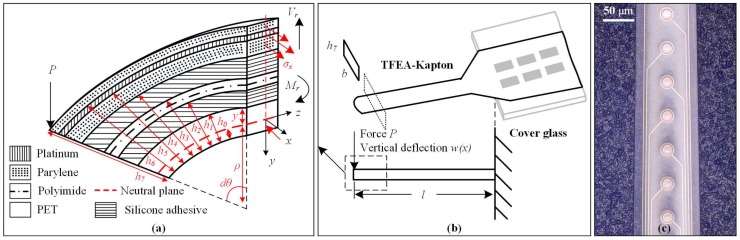
(**a**) Incremental element of a cantilever beam (dashed square in (**b**)); (**b**) Cantilever beam model based on the loading conditions in the bending experiment; (**c**) Optical microscopy of the TFEA’s patterned metal layer.

In the analytical model, the TFEA-Kapton was modeled as a laminate cantilever beam, with layers of PET, silicone adhesive, polyimide, silicone adhesive, Parylene C, Pt, and Parylene C, sequentially numbered with *i* = 1–7 from bottom to top, as shown in Figure 4a,b. The beam had a uniform width *b* = 800 μm, and a total length of *l* = 18.5 mm, which was the projected length of the 21-mm long TFEA-Kapton array in the *xz*-plane. The thickness of different layers is listed in Table 1 [17]. The material properties of PET [19], silicone adhesive [20], polyimide [19,21], Parylene C [22], and Pt are listed in Table 2, respectively (all material properties unreferenced are from the Comsol Multiphysics built-in material library). For simplification, the metal layer of TFEA was modeled as a uniform layer of Pt with a thickness of 200 nm covering the whole substrate, while the actual metal layer of TFEA consisted of patterned 20-nm-thick Ti and 200-nm-thick Pt. The actual area of the metal layer was less than 50% the whole area of the substrate as shown in Figure 4c.

Due to the large “length-to-thickness” ratio of the TFEA-Kapton (*l*:*h*_7_ = 105), the following assumptions were made to balance computation accuracy and complexity.

(1) The planar assumption: Plane sections before bending remain plane after bending. This assumption neglects the sheer stress *V_r_* on the transverse plane. The load, in this case, is such that no twisting occurred.
(2) The constant transverse plane assumption: All longitudinal elements have the same length, transverse surface, and material properties.
(3) The neutral plane continuity assumption: The neutral plane of the laminate remains continuous with no change in length when bending.

For a given incremental element in Figure 4a, when only one layer is studied, the deformation geometry is as follows:

*ε_x_* = [(*y* + *ρ*)*dθ* − *ρdθ*]/*ρdθ* = *y*/*ρ* (1)

Here,

1/*ρ* ≈ *w(x)*’’ (2)

where *ε* is the normal strain at distance *y* from the neutral axis, *ρ* is the radius of curvature of the neutral axis, *dθ* is the angle between the cross-sectional sides of the incremental element, and *w(x)* is the deflection function along the *y*-axis. Equation (2) is an approximation by neglecting the high order term of (*dy/dx*)^2^.

For the physical correlation, the elastic-range normal stress in the transverse plane follows the generalized Hooke’s law:

*ε_x_* = [*σ_x_* − *v*(*σ_y_* + *σ_z_*)]/*E*

Given the planar assumption, the bending state can be simplified to a uniaxial stress state:

*σ_x_* = *Eε_x_* (3)

where *σ_x_* is uniformly distributed in the *z*-direction while varying linearly in the *y*-direction, *σ_z_* is zero throughout the beam due to the absence of *z*-direction load, and *σ_y_* is neglected according to the planar assumption.

For the static force and moment equilibrium equations:

Σ*F_x_* = 0: ∫*_A_ σ_x_dA* = 0 (4)

*ΣM_z_* = 0: ∫*_A_ σ_x_ydA* = *M_r_* = *P(x* − *l)* (5)

where *A* is the sectional surface area, *M_r_* is the moment resulted from the *σ_x_* of the transverse plane, *P* is the load at the center of the edge, and *x* is the distance from the fixed edge to the transverse plane.

The deflection equation is obtained by substituting Equations (1) and (3) into Equation (5):

1/*ρ* = *M_r_*/*EI_z_* (6)

where *I_z_* is the moment of inertia of *z*-axis and equals ∫*_A_ y*^2^*dA*. Substituting Equation (2) into Equation (6) and rearranging gives

*EI_z_*∙*w*(x)’’ ≈ *M_r_* (7)

The deflection function *w*(*x*) was deducted by double integration with the constant transverse plane assumption, meaning *E* and *I_z_* remain constant throughout the length. *EI_z_* is taken as the bending stiffness. To agree with the bending experiment, the boundary conditions were a fixed end at one edge (*w*(*x* = 0) = 0, *w*′(*x* = 0) = 0) and a concentrated load at the other edge (*F*(*x* = 0) = *P*), which gives

*EI_z_*∙*w*(*l*) = −*Pl*^3^/3 (8)

where *w*(*l*) is the deflection of the loading point.

Furthermore, the deflection of the whole multi-layer laminate beam in this work can be calculated with the following sequence:

(1) List the design parameters for each layer including Young’s modulus *E* and layer position *h*:

*E* = (*E*_1_, *E*_2_, *E*_3_, *E*_4_, *E*_5_, *E*_6_, *E*_7_)

*h* = (*h*_0_, *h*_1_, *h*_2_, *h*_3_, *h*_4_, *h*_5_, *h*_6_, *h*_7_)

where *h*_0_ determines the position of the neutral layer. The other parameters are listed in Table 2.

(2) Determine *h*_0_ with Equations (1), (3) and (4):

(9) $$\sum_{i=1}^{7}\left(\int_{h_{i-1}}^{h_{i}}\left[E_{i}\left(y-h_{0}\right)\cdot bdy/\rho \right]\right)=0$$

(3) Determine equivalent bending stiffness with Equation (5):

(10) $$\sum_{i=1}^{7}\left(\int_{h_{i-1}}^{h_{i}}\left[E_{i}{\left(y-h_{0}\right)}^{2}\cdot bdy/\rho \right]\right)=P(x-1)$$

Thus,

$$1/\rho =P(x-l)/\sum_{i=1}^{7}\left(\int_{h_{i-1}}^{h_{i}}\left[E_{i}{\left(y-h_{0}\right)}^{2}\cdot bdy\right]\right)$$

Similar to Equation (6), we use the following formula as the equivalent bending stiffness (*EI*)*_e_*:

(11) $$\sum_{i=1}^{n}\left(\int_{h_{i-1}}^{h_{i}}\left[E_{i}{\left(y-h_{0}\right)}^{2}\cdot bdy\right]\right)={\left(EI\right)}_{e}$$

where *n* represents the number of layers and was 7 in the present work.

From Equation (8), within the elastic deflection range, when one edge of the beam was fixed and a perpendicular force *P* was applied to the center of the other edge, the deflection at the loading point *w*(*l*) was calculated with (*EI*)*_e_*:

(*EI*)*_e_*∙*w*(*l*) = −*Pl*^3^/3 (12)

**Figure 5:**
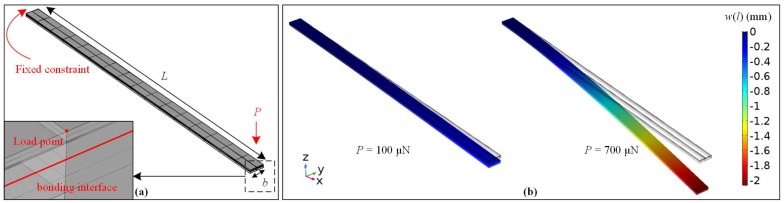
(**a**) Finite element model (FEM) with mesh rendering. (**b**) *w*(*l*) at the loading point with the increase of load *P*.

Alternatively, the multi-layer laminate beam was also modeled in an FEM with the solid mechanic module of Comsol Multiphysics 5.3a (Comsol AB, Stockholm, Sweden). With the same geometrical, material, and physical inputs as the analytical model, the *w*(*l*) at the loading point was also calculated. The FEM was built with 7 pairs of block elements stacking up from bottom to top, representing the 7 layers of TFEA-Kapton. Young’s modulus, Poisson’s ratio, and density were chosen from Table 2, among which Young’s modulus was kept the same between the analytical model and FEM. The whole model was selected as the isotropic linear elastic material. Figure 5a shows the physic settings of the FEM, which agreed with the bending experiment in this work. A fixed constraint was added to all the *yz*-plane faces at one end (*x* = 0), while a perpendicular point load *P* was added to the center of the top edge at the other end (*x* = *l*). A contact pair with adhesion attribute was added to the bonding interface, the tensile strength was set to be 79 kPa [17]. The meshing element size of the FEM was set to be 0.518–2.78 mm. A swept was employed from the top surface to the bottom surface of the FEM, with distribution nodes added to the platinum and top Parylene layer across their much thinner structures. With a stationary solver, a parametric sweep was conducted for *P* from 25 μN to 700 μN with a step of 25 μN. Figure 5b shows the vertical deflection *w*(*l*) of the loading point as a function of *P*.

**Figure 6:**
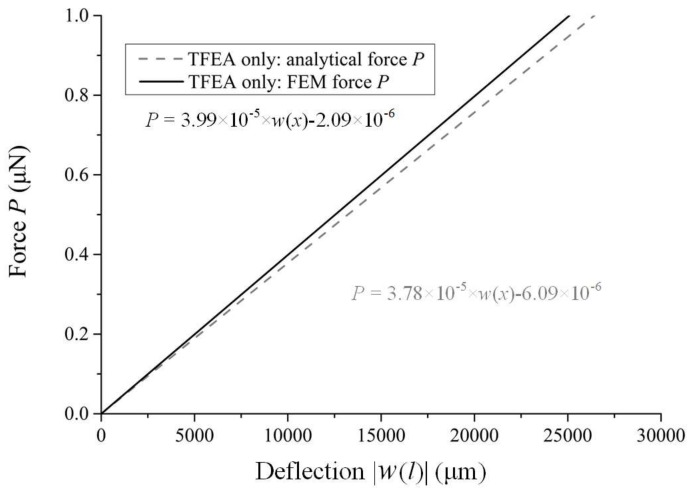
Deflection |*w*(*l*)| at the loading point of the TFEA with the increase of load *P* calculated with the analytical and FEM, respectively.

As an example, for the TFEA only, when *P* increased from 0 μN to 1 μN, Figure 6 shows the linear positive correlation between *P* and |*w*(*l*)|, from which the (*EI*)*_e_* of TFEA was found to be ~7 × 10^−11^ N∙m^2^. The calculation deviation between the analytical model and the FEM was ~5.6%. The analytical results generally agreed with the FEM results.

### 3.2. Bending Experiment

**Figure 7:**
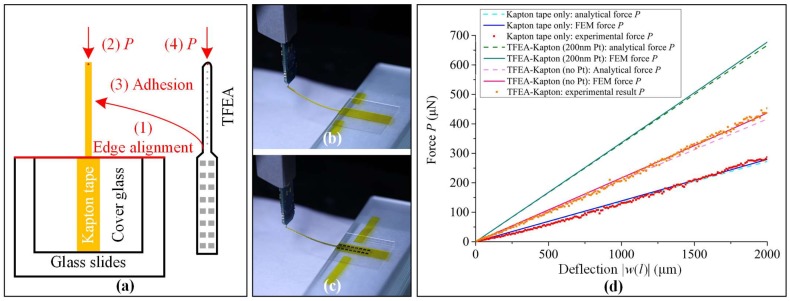
(**a**) Schematic of the bending experiment; (**b**) bending experiment of the Kapton tape; (**c**) bending experiment of the TFEA-Kapton; (**d**) deflection force *P* as a function of the vertical deflection |*w*(*l*)| at the loading point, in comparison to the analytical and FEM results.

A cantilever beam bending experiment was conducted to measure the bending stiffness of the Kapton tape and the TFEA-Kapton. Figure 7a shows the schematic of the experimental protocol: (1) A Kapton tape was first manually carved and adhered to the back of a piece of cover glass. It was then fixed onto a stack of glass slides with one edge of both the cover glass and the glass slide being aligned; (2) A force sensor probe (FT-S 100 μN Microforce Sensing Probe, FemtoTools AG, Buchs, Switzerland) with a precision of 0.05 μN was vertically mounted onto a micromanipulator. Figure 7b shows that a vertical bending force was applied to the center of the Kapton tape’s apical end. The deflection force *P* as a function of the vertical deflection |*w*(*l*)| was measured by the force probe. The range of |*w*(*l*)| was set to be 0–2000 μm with a step of 25 μm; (3) The Kapton tape was first detached from the cover glass and then used to make the TFEA-Kapton following the preparation procedures in Section 2; (4) Figure 7c shows that another bending experiment was repeated for the TFEA-Kapton adopting the same method as Procedure (2).

Figure 7d lists the loading point *P*-|*w*(*l*)| function from the experiment, the analytical model, and the FEM. The experimental *P*-|*w*(*l*)| results showed identifiable linearity, meaning the measured deflection range of 0–2000 μm was within the elastic range of deflection. The (*EI*)*_e_* from each result was obtained with Equation (12) after linear fitting. Table 3 shows that the Kapton tape had an (*EI*)*_e_* of 3.10 × 10^−7^ N∙m^2^ from the experimental result. The analytical model and FEM produced close (*EI*)*_e_* results compared to the experiment, with a deviation of −7.31% and −2.63%, respectively. The TFEA-Kapton had an (*EI*)*_e_* of 4.96 × 10^−7^ from the experimental result. The analytical model and FEM had a deviation of 41.77% and 44.07% for the calculation of TFEA-Kapton’s (*EI*)*_e_*, respectively. The calculation error mainly came from the model of the metal layer. The simplified 200-nm-thick Pt layer employed in the analytical model and FEM significantly enlarged the actual area of the metal layer, which was appropriate to act as the upper limit of (*EI*)*_e_*. Correspondingly, to find the lower limit of (*EI*)*_e_*, the TFEA-Kapton with no Pt layer but a uniform 6-μm-thick Parylene C was modeled and had a calculation deviation of −11.44% from the analytical model and −6.85% from the FEM, respectively. The experimental (*EI*)*_e_* approximately laid in between the upper and lower limit of the calculated (*EI*)*_e_* and was much closer to the lower limit. This corresponded to the fact that the patterned metal layer had a relatively small area, compared with the whole area of the substrate.

## 4. Discussion

The TFEA-Kapton was proven to mechanically enhance the TFEA. The TFEA-Kapton had an (*EI*)*_e_* of 4.96 × 10^−7^ from the experimental result, which was at least 10^3^ times higher than the TFEA’s (*EI*)*_e_* (~7 × 10^−^^11^ N∙m^2^ from the calculation). The enhancement was higher than the 600% bending stiffness increase of the structural TFEA design from Johnson and Wise [7]. On the other hand, the TFEA, in turn, stiffened the Kapton tape significantly after adhering to it. With a thickness being ~4.5% ((5 + 0.2 + 1) μm/(75 + 35 + 25 + 35) μm = 4.5%) and an (*EI*)*_e_* being ~10^−3^ of the Kapton tape, the TFEA increased the (*EI*)*_e_* of the Kapton tape by 60% after adhering to it.

Such carrier-aided TFEA designs have potentials and challenges in terms of further development and commercial application. Carrier-aided TFEA designs have several advantages: (1) They fully utilize the throughput of the TFEA’s batch-processed micro-fabrication method, reducing cost and labor intensity; (2) Since TFEA carriers do not involve any electronic component, the fabrication of TFEA carriers is much easier than assembling the commercial cochlear electrode array; (3) The utilization of TFEA carriers provides easy access to the readily available CI insertion techniques including “advance off-stylet,” eliminating intricate fabrication steps for the TFEA [7]. Specifically, Kapton tape has potentials for future CI TFEA carriers because (1) it can further be made via a laser-cut method with a micrometer-level precision [23] and (2) it has been proven biocompatible in a long-term implant study [24]. The main challenge of carrier-aided TFEA designs is the optimal design of the carrier geometry and flexibility, which should be stiff enough to be inserted and flexible enough to avoid insertion trauma. From the present work, the Parylene TFEA with a much thinner thickness (4.5%) and greater flexibility (~1‰ bending stiffness) significantly changed the bending stiffness (increased by 60%) after adhering to the carrier. This finding suggests that the TFEA should also be included for the design of the carrier in terms of geometry and material selection. The analytical model and FEM in this work can serve as a preliminary estimation of the mechanical design of the carrier-aided TFEA.

As a proof-of-concept study, the present TFEA-Kapton has several limitations and can be improved in the following directions: (1) Kapton tape was selected as a case study for its insulativity, cost-effectiveness, planar geometry, and upward pre-bending shape. Long-term biocompatibility, stability, and insertion precision should be considered in the future design of TFEA carriers; (2) The equivalent bending stiffness models in the present work were based on the multi-layer laminate beam, which only applied to planar-shaped carriers and simplified the metal layer. Modifications are needed for cylindrical-shaped carriers and other designs.

## 5. Conclusions

In this study, we characterized the mechanical properties of a carrier-aided thin film electrode array design with a Parylene TFEA adhered onto a Kapton tape. The equivalent bending stiffness of the TFEA and the TFEA-Kapton were further investigated with an analytical model, an FEM, and a cantilever bending experiment, severally. The results demonstrated that the Parylene TFEA with a 4.5% thickness and ~1‰ bending stiffness of the Kapton carrier increased the bending stiffness of the Kapton carrier by 60% after adhering to it. The proof-of-concept study suggests that the TFEA should not be ignored in the design of its carrier to realize the desired mechanical strength for cochlear insertion.

## Figures and Tables

**Table 1:** Thickness and *y*-axis location of different layers.

Layer Material	PET (*i* = 1)	Bottom Silicone Adhesive (*i* = 2)	Polyimide (*i* = 3)	Top Silicone Adhesive (*i* = 4)	Bottom Parylene (*i* = 5)	Platinum (*i* = 6)	Top Parylene (*i* = 7)
*y*-axis location (μm) (*h_i_*)	75	110	135	170	175	175.2	176.2

**Table 2:** Material properties of different layers.

Layer Material	PET (*i* = 1)	Silicone Adhesive (*i* = 2 or 4)	Polyimide (*i* = 3)	Parylene C (*i* = 5 or 7)	Platinum (*i* = 6)
Young’s modulus (*E_i_*) (GPa)	2.10	4.15 × 10^−^^3^	2.07	2.80	154
Density (*D_i_*) (kg/m^3^)	1430	1280	1420	1289	21,387
Poisson’s ratio (*ν_i_*)	0.40	0.49	0.35	0.40	0.345

**Table 3:** Equivalent bending stiffness results.

Object of Study	Method	Equivalent Bending Stiffness (*EI*)*_e_* (N∙m^2^)	Calculation Deviation Compared with Experimental Results
Kapton tape only	Analytical model	2.88 × 10^−7^	−7.31%
	FEM	3.02 × 10^−7^	−2.63%
	Experiment	3.10 × 10^−7^	N/A
TFEA-Kapton	Analytical model with 200-nm-thick Pt	7.03 × 10^−7^	41.77%
	FEM with 200-nm-thick Pt	7.14 × 10^−7^	44.07%
	Analytical model with no Pt	4.39 × 10^−7^	−11.44%
	FEM with no Pt	4.62 × 10^−7^	−6.85%
	Experiment	4.96 × 10^−7^	N/A
